# Exposure to micro‐ and nano‐plastics: From human internal burden to systems‐level health‐risk interpretation

**DOI:** 10.1002/imt2.70156

**Published:** 2026-07-28

**Authors:** Chen Tu, Jie Yang, Jingyi Liao, Rongkui Hu, Yudong Feng, Di Wu, Yankai Xia, Dan Wang, Jinrong Tang, Xianzheng Yuan, Dong Zhu, Jing Wei, Li Xu, Mingkai Xu, Xuetao Guo, Jian Zhao, Qiqing Chen, Zhenming Zhang, Lianzhen Li, Yini Ma, Xiaoxu Jiang, Kai Zhang, Cheng Peng, Mengyao Li, Ning Shen, Willie J. G. M. Peijnenburg, Matthias C. Rillig, Jason C. White, Yongming Luo, Ningwei Zhao

**Affiliations:** ^1^ State Key Laboratory of Soil and Sustainable Agriculture Institute of Soil Science, Chinese Academy of Sciences Nanjing China; ^2^ Affiliated Hospital of Nanjing University of Chinese Medicine Nanjing China; ^3^ State Key Laboratory of Reproductive Medicine and Offspring Health, Center for Global Health, School of Public Health Nanjing Medical University Nanjing China; ^4^ College of Resources and Environmental Sciences Nanjing Agricultural University Nanjing China; ^5^ Shandong Key Laboratory of Synergistic Control of Complex Multi‐Media Pollution, School of Environmental Science and Engineering Shandong University Qingdao China; ^6^ State Key Laboratory of Regional and Urban Ecology, Ningbo Observation and Research Station, Institute of Urban Environment Chinese Academy of Sciences Xiamen China; ^7^ Key Laboratory of Soil Environment Management and Pollution Control Nanjing Institute of Environmental Sciences, Ministry of Ecology and Environment of China Nanjing China; ^8^ Institute of Quality Standard and Testing Technology Beijing Academy of Agriculture and Forestry Sciences Beijing China; ^9^ Key Laboratory of Pollution Ecology and Environmental Engineering Institute of Applied Ecology, Chinese Academy of Sciences Shenyang China; ^10^ College of Natural Resources and Environment Northwest A&F University Yangling China; ^11^ Institute of Coastal Environmental Pollution Control, Key Laboratory of Marine Environment and Ecology, Ministry of Education, Frontiers Science Center for Deep Ocean Multispheres and Earth System Ocean University of China Qingdao China; ^12^ State Key Laboratory of Estuarine and Coastal Research East China Normal University Shanghai China; ^13^ College of Resources and Environmental Engineering Guizhou University Guiyang China; ^14^ School of Environment and Geography, Shandong Key Laboratory of Synergistic Control of Complex Multi‐Media Pollution Qingdao University Qingdao China; ^15^ Key Laboratory of Agro‐Forestry Environmental Processes and Ecological Regulation of Hainan Province, School of Environmental Science and Engineering Hainan University Haikou China; ^16^ China National Environmental Monitoring Centre Beijing China; ^17^ National Observation and Research Station of Coastal Ecological Environments in Macao, Macao Environmental Research Institute Macau University of Science and Technology Macao China; ^18^ Key Laboratory of Environmental Risk Assessment and Control on Chemical Process, Ministry of Ecology and Environment, School of Resource and Environmental Engineering East China University of Science and Technology Shanghai China; ^19^ State Key Laboratory of Systems Medicine for Cancer, Shanghai Cancer Institute, Renji Hospital Shanghai Jiao Tong University School of Medicine Shanghai China; ^20^ Nantong 4th People's Hospital Kangda College of Nanjing Medical University Affiliated Nantong Mental Health Center Nantong China; ^21^ China Exposomics Institute Shanghai China; ^22^ National Institute of Public Health and the Environment Center for the Safety of Substances and Products Bilthoven the Netherlands; ^23^ Institute of Biology Freie Universität Berlin Berlin Germany; ^24^ The Connecticut Agricultural Experiment Station New Haven USA

## Abstract

Micro‐ and nano‐plastics (MNPs) can enter the human body through inhalation, ingestion, contact with consumer products, and iatrogenic routes. The clinical relevance of MNP exposure should be interpreted at three levels: barrier interaction, cellular stress, and system‐level disruption, with key mechanisms including oxidative stress, mitochondrial dysfunction, and chronic inflammation.

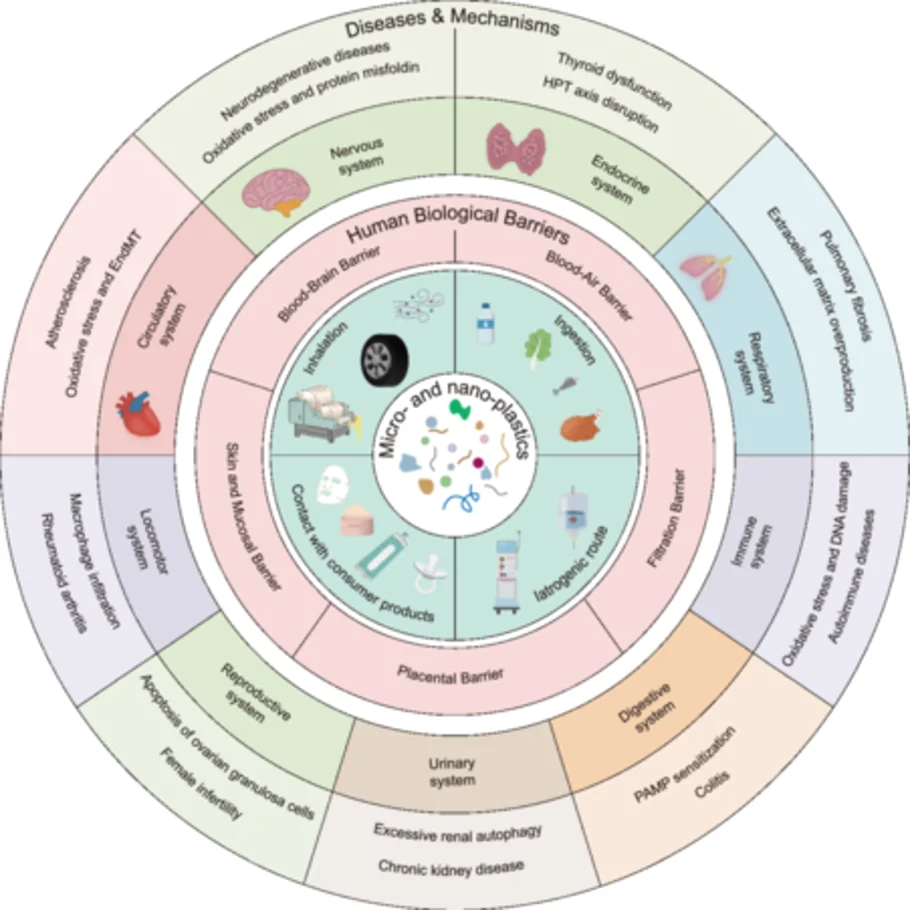

To the Editor,

Microplastics (MPs) are generally defined as plastic particles smaller than 5 mm, whereas nanoplastics (NPs) are typically defined as plastic particles smaller than 1 μm. Micro‐ and nano‐plastics (MNPs) can persist, migrate, age, and interact with plastic‐associated additives and co‐contaminants in soil, water, sediments, air, and biota [1, 2, 3]. Their ubiquitous presence leads to persistent human exposure. For health assessment, however, the central question is no longer only whether MNPs can be detected in humans, but whether specific exposure conditions generate a biologically significant internal burden.

Recent studies indicate that MNPs can be detected in non‐organ biological samples and in tissue‐ and organ‐associated compartments beyond initial exposure interfaces. Human exposure may occur through inhalation, ingestion, contact with consumer products, and iatrogenic routes [4, 5, 6, 7]. However, the strength of evidence differs markedly among organ systems. Cardiovascular findings are currently the most clinically informative because particles have been detected in vascular lesions and correlated with prospective clinical outcomes, whereas reproductive, respiratory, neurological, endocrine, immune, urinary, ocular, and musculoskeletal findings remain more heterogeneous and often rely on observational or mechanistic evidence [4, 8, 9, 10, 11, 12]. Mechanistic studies suggest that mitochondrial dysfunction, oxidative stress, barrier disruption, inflammatory signaling, endocrine perturbation, and gut microbiota dysbiosis may contribute to MNP‐induced toxicity [3, 11, 12, 13, 14, 15, 16], but direct causal inference in humans remains limited.

In this Correspondence, we propose internal burden as a bridging metric that links external exposure, organ‐specific distribution, mechanistic perturbation, and potential clinical relevance. By integrating exposomics, microbiome‐centered mechanisms, multi‐omics, and organ‐specific internal burden, we aim to clarify the hierarchy of existing human evidence, identify methodological limitations, and propose practical research priorities to shift research focus from mere detection to quantitative and clinically valid interpretation of MNP exposure.

## ROUTES OF HUMAN EXPOSURE TO MNPS

**Figure 1:**
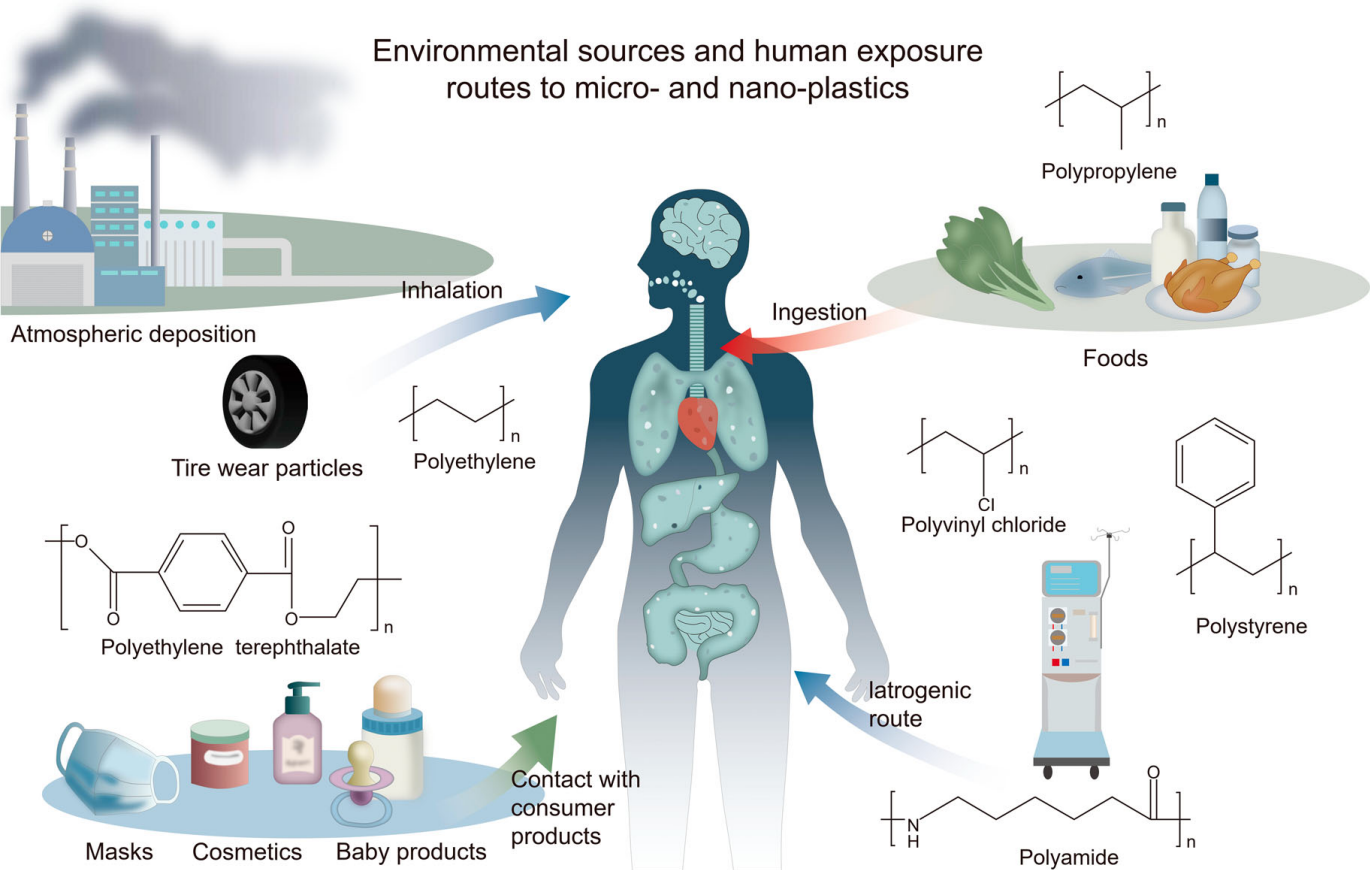
Environmental sources and human exposure routes to micro‐ and nano‐plastics (MNPs). Micro‐ and nano‐plastics are released from diverse natural and anthropogenic sources and can enter the human body via multiple exposure routes. Inhalation exposure may arise from airborne MNPs associated with atmospheric deposition, tire wear particles, and occupational settings like textile‐processing facilities. Ingestion constitutes another dominant exposure route, with MNPs detected in drinking water, milk, vegetables, crops, fish, poultry, and other foods. Direct contact with consumer products, including face masks, cosmetics or facial cleansers containing microbeads, and infant products like pacifiers, may also contribute to human exposure. In addition, iatrogenic exposure may arise from medical procedures and devices, such as intravenous infusion systems and dialysis equipment. Polymer types identified in human biological matrices mainly consist of polyethylene (PE), polypropylene (PP), polystyrene (PS), polyethylene terephthalate (PET), polyamide (PA), and polyvinyl chloride (PVC).

Human exposure to MNPs is now recognized as continuous, cumulative, and heterogeneous (Figure 1). Inhalation remains one of the best‐established routes. Airborne MNP burden varies with traffic intensity, road and tire wear, built‐environment characteristics, and climate conditions [4]. Occupational contexts, such as textile manufacturing, plastic production, and waste processing, may generate higher exposure due to higher particle concentrations in occupational ambient air. Inhaled MNPs may also coexist with combustion‐related pollutants and traffic‐derived contaminants, which could amplify pulmonary and systemic responses. Thus, inhalation should be viewed as a prolonged and biologically relevant exposure route, especially in urban and occupational contexts.

Ingestion may represent a major chronic exposure route for the general population. MNPs can enter the food chain via crops, vegetables, drinking water, as well as trophic transfer within terrestrial and aquatic food webs [5, 6]. This route is particularly relevant for submicron and nanoplastic fractions, which may show greater mobility, treatment‐barrier penetration, and bioavailability after ingestion. For example, available exposure estimates suggest that dietary and drinking‐water intake may reach hundreds of particles per day under common exposure scenarios, although values differ markedly among studies [5]. Nevertheless, dietary exposure estimates vary substantially across matrices, particle‐size cutoffs, sampling strategies, and analytical methods. Oral exposure should therefore be interpreted as a long‐term and multifaceted process rather than a single quantitative intake value.

Contact with consumer products constitutes another important exposure scenario. MNPs have been identified or implicated in cosmetics, microbead‐containing toothpaste, electronic cigarette liquids and aerosols, sanitary products, face masks, plastic tableware, and baby products such as bottles and silicone teats [4]. For example, simulated use of plastic baby bottles and silicone teats has been reported to release measurable numbers of MNPs, indicating that daily consumer products may contribute to oral or mucosal exposure [4]. This category should be viewed as an exposure scenario rather than a distinct biological entry route, because released particles may contact the skin, oral cavity, respiratory tract, ocular surface, and other mucosal interfaces. Therefore, consumer‐product‐related exposure is not confined to the gastrointestinal tract and may involve multiple biological barriers.

Beyond conventional exposure routes, MNPs may also enter the human body through iatrogenic exposure scenarios, including infusion systems, intravenous therapy, and cardiopulmonary bypass circuits [4]. This route merits special attention because it can bypass epithelial barriers and introduce particles directly into circulation. For example, simulated intravenous therapy released polypropylene (PP)‐, polyethylene (PE)‐, and polyvinyl chloride (PVC)‐derived MNPs at (5.82 ± 0.86) × 10^4^ particles/L, with particle sizes ranging from 10 nm to 3.6 μm [7]. Such exposure may be particularly relevant for children, critically ill patients, and individuals receiving repeated or prolonged medical interventions. From an exposomic perspective, these diverse routes highlight the need for integrative exposure measures that can be linked to internal burden and downstream molecular responses.

## INTERNAL BURDEN OF MNPS

Internal burden refers to the quantity, composition, and biodistribution of MNPs retained within the human body after exposure. Current evidence can be broadly divided into two categories: (1) biological fluids or other non‐organ samples, including feces, urine, sputum, blood, bronchoalveolar lavage fluid, nasal lavage fluid, breast milk, meconium, thrombi, semen, follicular fluid, cerebrospinal fluid, and synovial fluid; (2) tissue‐ and organ‐associated compartments, including lung parenchyma, intestinal tissue, liver, spleen, kidney, placenta, vascular lesions, and ocular surface‐related tissues [3, 4, 16, 17]. Across these matrices, the predominant polymers include PE, PP, polystyrene (PS), polyethylene terephthalate (PET), PVC, and polyamide (PA) (Figure 1).

These findings suggest that at least some MNPs can move beyond initial exposure interfaces and reach multiple biological compartments. In the respiratory tract, fibrous particles are frequently detected in lung tissue and bronchoalveolar lavage fluid, possibly reflecting reduced mobility and prolonged airway retention. In the gastrointestinal tract, small particles, including submicron fractions, may cross the epithelial barrier via endocytosis, transcytosis or microfold cell‐related pathways and subsequently affect barrier integrity, epithelial transport, nutrient assimilation, and gut–systemic homeostasis. In highly perfused or clearance‐related organs, such as the liver, spleen, and kidneys, abundant blood flow and phagocytic or scavenger‐cell activity may contribute to particle retention. Detection in placental tissue and follicular fluid further suggests that reproductive and developmental microenvironments warrant particular attention [4, 17].

The heterogeneity of internal burden is shaped by four interrelated dimensions: particle characteristics, exposure context, host factors, and barrier integrity. Particle‐level determinants include size, shape, polymer type, surface charge, environmental aging, eco‐corona formation, and sorbed chemicals, all of which can alter the effective biological identity of MNPs. Exposure context includes route or scenario, frequency, duration, and dose, whereas host factors such as age, disease status, physiological state, and occupational intensity may influence particle uptake, clearance, and retention. Barrier integrity, including that of epithelial, endothelial, placental, renal, and other biological barriers, further determines how efficiently MNPs move beyond initial contact interfaces. Together, these factors influence trans‐barrier transport, tissue retention, and the measured burden in different biological compartments, helping explain why MNP burden is unlikely to be evenly distributed across organs, individuals, or studies. They also underscore the need for harmonized metrics for both particle number and polymer mass.

However, detection alone should not be equated with biologically active or disease‐relevant burden, because current analytical methods remain limited in their ability to distinguish transient MNP passage, long‐term tissue retention, and exposure levels sufficient to induce biologically significant effects. Consequently, the interpretation of human internal‐burden data remains constrained by methodological limitations. Current evidence relies largely on Raman spectroscopy, Fourier‐transform infrared (FTIR) spectroscopy, and pyrolysis gas chromatography–mass spectrometry (Py‐GC/MS), which differ in sensitivity, throughput, size resolution, and resistance to biological matrix interference [16]. Background contamination, variable digestion protocols, incomplete recovery, limited nanoparticle detection, and inconsistent particle‐ versus mass‐based reporting can produce false positives, false negatives, or non‐comparable estimates. Small particles, particularly NPs, remain difficult to detect, and macromolecules in biological samples, such as proteins, lipids, and other endogenous components, may mask, distort, or obscure particle signals. These issues compromise both mass‐based quantification and particle‐based assessments of MNPs. Therefore, current human data should be interpreted as evidence of plausible internal exposure rather than definitive quantitative body burden unless supported by rigorous blank controls, recovery tests, detection‐limit reporting, standardized protocols, and at least two orthogonal analytical methods. Integration with transcriptomic, metabolomic, microbiomic, and immunophenotyping data will be essential for distinguishing mere presence from biologically active exposure.

## CLINICAL RELEVANCE ACROSS ORGAN SYSTEMS

Current evidence suggests that the clinical relevance of MNP exposure should be interpreted according to an evidence hierarchy rather than assumed to be uniform across organ systems. Key criteria include exposure‐to‐burden plausibility, methodological reliability, mechanistic support, and the strength of human evidence. Within this hierarchy, cardiovascular disease currently provides the strongest human evidence signal. MNPs have been detected in carotid atheromas, and lesion‐associated particles have been prospectively linked to adverse cardiovascular outcomes [18]. Although these findings do not establish causality, they provide the clearest indication to date that internal particle burden may have prognostic significance.

Respiratory and reproductive findings are biologically plausible and increasingly suggestive, but remain less conclusive. Respiratory evidence is supported mainly by occupational studies linking exposure to PA, PE, PP, or PVC particulates with chronic airway inflammation, interstitial lung disease, and impaired pulmonary function [12]. Reproductive evidence includes associations between follicular‐fluid MP concentrations and diminished ovarian reserve, with higher total MPs reported in women with diminished ovarian reserve than in controls (30.63 vs. 18.48 μg/g) [17]. Detection in follicular and placental compartments further suggests that reproductive microenvironments and early developmental windows may be vulnerable, although prospective studies and mechanistic validation remain necessary [4, 13, 17].

**Figure 2:**
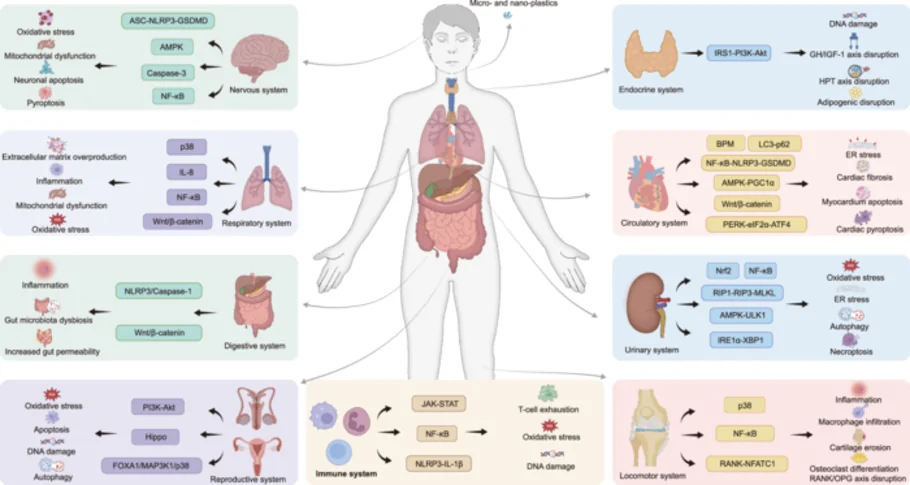
Disease risks and mechanisms of exposure to micro‐ and nano‐plastics in human organ systems. Micro‐ and nano‐plastics (MNPs) can translocate across initial exposure barriers and exert adverse effects on multiple organ systems, including the nervous, respiratory, digestive, reproductive, immune, endocrine, circulatory, urinary, and locomotor systems. Documented toxicological effects encompass oxidative stress, mitochondrial dysfunction, inflammation, DNA damage, apoptosis, pyroptosis, autophagy, endoplasmic reticulum (ER) stress, barrier impairment, gut microbiota dysbiosis, and immune or endocrine disruption. These perturbations are mediated by multiple signaling pathways, such as NF‐κB, PI3K‐Akt, and p38 signaling cascades. Collectively, these findings reveal shared and organ‐specific mechanisms mediating systemic toxicity elicited by MNPs.

For gastrointestinal, hepatic, metabolic, neurological, ocular, endocrine, urinary, immune, and musculoskeletal outcomes, the evidence remains emerging and should be framed cautiously (Figure 2 and Table S1). Reported associations with gut barrier dysfunction, dysbiosis, intestinal inflammation, hepatic steatosis, and metabolic disturbance support a possible gut‐centered pathway linking internal burden to disease susceptibility [9, 13, 14]. Additional signals include detection in tear fluid and meibum, as well as experimental or observational links to neuroinflammation, blood–brain barrier disruption, and neurodevelopmental toxicity [4, 19]. Across these outcomes, recurrent mechanistic themes include oxidative stress, mitochondrial dysfunction, inflammasome activation, autophagy imbalance, and NF‐κB‐related signaling [4, 8, 10].

Overall, current human evidence can be stratified into three levels: prospective or longitudinal clinical evidence, which is strongest for cardiovascular disease; cross‐sectional or observational associations, which are increasingly suggestive for respiratory and reproductive outcomes; and experimental, mechanistic, or exploratory evidence for other organ systems. Future studies should avoid inferring causality from detection alone. Instead, causal interpretation will require repeated internal‐burden measurements, longitudinal follow‐up of clinical outcomes, matched exposure and multi‐omics profiling, and triangulation across epidemiological, experimental, and mechanistic evidence.

## MECHANISTIC INSIGHTS INTO MNP‐INDUCED TOXICITY

Mechanistic investigations are beginning to explain why MNP exposure may affect multiple organ systems, but these findings must be interpreted in light of variation in concentration, exposure context, and biological models. Oxidative stress, mitochondrial dysfunction, cytotoxicity, genotoxicity, and chronic inflammatory signaling remain central mechanisms [3, 11, 14]. However, the magnitude and relevance of these responses depend on particle size, polymer type, shape, aging status, surface chemistry, digestive transformation, exposure duration, and the combined effects of additives and sorbed co‐contaminants. Because experimental concentrations vary widely across studies, reported mechanistic effects should be regarded as model‐ and context‐specific observations rather than direct thresholds for human disease.

These context‐dependent effects can be understood as a progression from particle identity to barrier interaction, cellular stress, and system‐level network disruption. Particle properties and associated chemicals first define the biological identity of MNPs and influence their interaction with host tissues. At biological interfaces, events at the intestinal, pulmonary, placental, blood–brain, and reproductive barriers determine translocation, local retention, and tissue injury. At the cellular level, mitochondrial damage, oxidative injury, inflammasome activation, autophagy imbalance, NF‐κB/MAPK activation, FOXA1/p38 signaling, and defective efferocytosis connect particle exposure to tissue dysfunction [10, 11, 20]. At the systems level, microbiome–immune–metabolic axes, including gut–liver, gut–brain, and gut–reproductive interactions, may translate local perturbations into multi‐organ outcomes.

The growing support for a microbiota‐centered framework is especially relevant to *iMeta*. Chronic MNP exposure may reshape host‐associated microbial communities, microbial metabolites, extracellular vesicle signaling, immune tone, and barrier function, thereby linking environmental contamination and food‐chain exposure to systemic inflammatory, metabolic, reproductive, and developmental outcomes [13, 15]. Integrative multi‐omics approaches combining metagenomics, metabolomics, host transcriptomics, proteomics, immunophenotyping, and exposome assessment can help reconstruct causal networks connecting MNP burden to systems‐level dysfunction. Coupled with experimental validation, such approaches may move the field beyond descriptive toxicity toward interpretable biological networks.

## FUTURE DIRECTIONS

Future studies should first improve environmental relevance. Most human exposure to MNPs is chronic, low‐dose, multi‐route, and chemically complex, whereas many toxicological models still rely on short‐term, high‐dose, pristine, single‐polymer exposures. More representative models should incorporate environmentally aged particles, submicron and nanoplastic fractions, and mixture systems containing plasticizers, bisphenols, flame retardants, metals, and organic pollutants. Because aged or biofilm‐associated particles may interact differently with host‐associated microbiomes than pristine particles, microbiome‐informed exposure models will be particularly important for One Health interpretation. In addition, compared with animal models, human‐derived organoids can better recapitulate the physiological characteristics of human organs and precisely characterize MNP‐induced toxicity. Meanwhile, integrated multi‐organoid systems enable systematic investigation of inter‐organ toxicological crosstalk, such as that along the gut‐liver‐brain axis. Therefore, organoid‐based exposure models may represent a novel and reliable platform for elucidating the toxicological mechanisms underlying environmentally realistic MNP exposure.

A second priority is to develop quantitative exposure–internal burden–effect frameworks. Standardized internal‐burden assessment should report both particle number and polymer mass, together with size distribution, polymer identity, morphology, detection limits, recovery rates, and contamination controls. Longitudinal cohorts should combine repeated measurements of external exposure and internal burden with organ‐specific sampling, clinical phenotyping, and matched metagenomic, metabolomic, transcriptomic, proteomic, and immunophenotyping profiles. These datasets would allow dose–response modeling, causal mediation analysis, mixed‐effects models, and physiologically based toxicokinetic/toxicodynamic approaches to link exposure, internal burden, molecular perturbation, and clinical outcomes.

Within this broader framework, research priorities should be explicitly tiered according to evidence strength and translational urgency. Cardiovascular disease should be regarded as the strongest current human evidence base and prioritized for risk prediction and mechanistic validation. Reproductive and early‐life health, including sperm quality, ovarian reserve, placental transfer, fetal development, and transgenerational or epigenetic outcomes, represents a high‐priority translational domain. Respiratory outcomes also warrant continued attention because of their occupational relevance. Neurological, endocrine, immune, urinary, ocular, and musculoskeletal outcomes should be considered emerging areas that require stronger exposure assessment, longitudinal evidence, and mechanistic validation before firm clinical conclusions can be drawn.

In conclusion, MNP research has entered a stage in which human detection, internal burden, organ‐specific distribution, and biological responses need to be interpreted through a systems‐level framework. The field has moved beyond proof of presence, but direct causal inference in humans remains limited. The central challenge is now to determine which exposure conditions, internal‐burden ranges, and microbiome–immune–metabolic networks are sufficient to make MNP exposure biologically and clinically significant. Addressing this challenge will require harmonized analytical methods, environmentally realistic exposure models, longitudinal human studies, quantitative exposure–effect modeling, and close integration of exposure science with clinically grounded systems biology.

## AUTHOR CONTRIBUTIONS

**Chen Tu:** Conceptualization; writing—original draft; writing—review and editing; visualization; funding acquisition. **Jie Yang:** Writing—original draft; writing—review and editing. **Jingyi Liao:** Visualization; writing—review and editing. **Rongkui Hu:** Writing—review and editing. **Yudong Feng:** Writing—review and editing. **Di Wu:** Writing—review and editing. **Yankai Xia:** Writing—review and editing. **Dan Wang:** Writing—original draft; visualization. **Jinrong Tang:** Writing—original draft; visualization. **Xianzheng Yuan:** Writing—review and editing. **Dong Zhu:** Writing—review and editing. **Jing Wei:** Writing—review and editing. **Li Xu:** Writing—review and editing. **Mingkai Xu:** Writing—review and editing. **Xuetao Guo:** Writing—review and editing. **Jian Zhao:** Writing—review and editing. **Qiqing Chen:** Writing—review and editing. **Zhenming Zhang:** Writing—review and editing. **Lianzhen Li:** Writing—review and editing. **Yini Ma:** Writing—review and editing. **Xiaoxu Jiang:** Writing—review and editing. **Kai Zhang:** Writing—review and editing. **Cheng Peng:** Writing—review and editing. **Mengyao Li:** Writing—review and editing. **Ning Shen:** Writing—review and editing. **Willie J. G. M. Peijnenburg:** Writing—review and editing. **Matthias C. Rillig:** Writing—review and editing. **Jason C. White:** Writing—review and editing. **Yongming Luo:** Conceptualization; writing—original draft; writing—review and editing; funding acquisition. **Ningwei Zhao:** Conceptualization; writing—original draft; writing—review and editing; visualization; funding acquisition. All authors have read the final manuscript and approved it for publication.

## CONFLICT OF INTEREST STATEMENT

The authors declare no conflicts of interest.

## ETHICS STATEMENT

No animals or humans were involved in this study.

## Supporting information


**Table S1**: Disease risks and mechanisms of exposure to micro‐ and nano‐plastics in human organ systems.

## Data Availability

No new data were generated in this review. Supplementary materials (tables, graphical abstract, slides, videos, Chinese translated version and update materials) may be found in the online DOI or iMeta http://www.imeta.science/.
