# Supplementary material for: Exposure to micro‐ and nano‐plastics: From human internal burden to systems‐level health‐risk interpretation
Source: Imeta. 2026 Jul 28;5(4):e70156. doi: 10.1002/imt2.70156 (PMC13410800; doi:10.1002/imt2.70156)
Supplement: Supplementary file 1 [file IMT2-5-e70156-s001.docx]

**Supporting Information to**

**Exposure to micro- and nano-plastics: From human internal burden to systems-level health-risk interpretation**

**Running title:** Systems-level interpretation of micro- and nano-plastic exposure

Chen Tu^1^, Jie Yang^1^, Jingyi Liao^2^, Rongkui Hu^2^, Yudong Feng^1^, Di Wu^3^, Yankai Xia^3^, Dan Wang^1^, Jinrong Tang^4^, Xianzheng Yuan^5^, Dong Zhu^6^, Jing Wei^7^, Li Xu^8^, Mingkai Xu^9^, Xuetao Guo^10^, Jian Zhao^11^, Qiqing Chen^12^, Zhenming Zhang^13^, Lianzhen Li^14^, Yini Ma^15^, Xiaoxu Jiang^16^, Kai Zhang^17^, Cheng Peng^18^, Mengyao Li^19^, Ning Shen^20, 21^, Willie J. G. M. Peijnenburg^22^, Matthias C. Rillig^23^, Jason C. White^24^, Yongming Luo^1*^, Ningwei Zhao^2, 21*^

^1^State Key Laboratory of Soil and Sustainable Agriculture, Institute of Soil Science, Chinese Academy of Sciences, Nanjing 211135, China

^2^Affiliated Hospital of Nanjing University of Chinese Medicine, Nanjing 210029, China

^3^State Key Laboratory of Reproductive Medicine and Offspring Health, Center for Global Health, School of Public Health, Nanjing Medical University, Nanjing 211166, China

^4^College of Resources and Environmental Sciences, Nanjing Agricultural University, Nanjing 211800, China;

^5^Shandong Key Laboratory of Synergistic Control of Complex Multi-Media Pollution, School of Environmental Science and Engineering, Shandong University, Qingdao 266200, China

^6^State Key Laboratory of Regional and Urban Ecology, Ningbo Observation and Research Station, Institute of Urban Environment, Chinese Academy of Sciences, Xiamen 361021, China

^7^Key Laboratory of Soil Environment Management and Pollution Control, Nanjing Institute of Environmental Sciences, Ministry of Ecology and Environment of China, Nanjing 210042, China

^8^Institute of Quality Standard and Testing Technology, Beijing Academy of Agriculture and Forestry Sciences, Beijing 100097, China

^9^Key Laboratory of Pollution Ecology and Environmental Engineering, Institute of Applied Ecology, Chinese Academy of Sciences, Shenyang 110016, China

^10^College of Natural Resources and Environment, Northwest A&F University, Yangling 712100, China

^11^Institute of Coastal Environmental Pollution Control, Key Laboratory of Marine Environment and Ecology, Ministry of Education, Frontiers Science Center for Deep Ocean Multispheres and Earth System, Ocean University of China, Qingdao 266100, China

^12^State Key Laboratory of Estuarine and Coastal Research, East China Normal University, Shanghai 200241, China

^13^College of Resources and Environmental Engineering, Guizhou University, Guiyang 550025, China

^14^School of Environment and Geography, Shandong Key Laboratory of Synergistic Control of Complex Multi-Media Pollution, Qingdao University, Qingdao 266071, China

^15^Key Laboratory of Agro-Forestry Environmental Processes and Ecological Regulation of Hainan Province, School of Environmental Science and Engineering, Hainan University, Haikou 570228, China

^16^China National Environmental Monitoring Centre, Beijing 100012, China

^17^National Observation and Research Station of Coastal Ecological Environments in Macao, Macao Environmental Research Institute, Macau University of Science and Technology, Macao 999078, China

^18^Key Laboratory of Environmental Risk Assessment and Control on Chemical Process, Ministry of Ecology and Environment, School of Resource and Environmental Engineering, East China University of Science and Technology, Shanghai 200237, China

^19^State Key Laboratory of Systems Medicine for Cancer, Shanghai Cancer Institute, Renji Hospital, Shanghai Jiao Tong University School of Medicine, Shanghai 200127, China

^20^Nantong 4th People's Hospital, Kangda College of Nanjing Medical University Affiliated Nantong Mental Health Center, Nantong 226005, China

^21^China Exposomics Institute, Shanghai 200120, China

^22^National Institute of Public Health and the Environment, Center for the Safety of Substances and Products, Bilthoven 3720BA, the Netherlands

^23^Institute of Biology, Freie Universität Berlin, Berlin 14195, Germany

^24^The Connecticut Agricultural Experiment Station, New Haven 06504, USA

^*^**Correspondence:** ymluo@issas.ac.cn (Yongming Luo), mass.zhao@exposomics-institute.com (Ningwei Zhao)

**Table S1 Disease risks and mechanisms of exposure to micro- and nano-plastics in human organ systems**

| **Organ systems** | **Typical diseases** | **Mechanisms** | **Signaling pathways** | **References** |
| --- | --- | --- | --- | --- |
| Nervous system | Neurodegenerative diseases | Oxidative stress; Pyroptosis; Mitochondrial dysfunction; Neuronal apoptosis | ASC-NLRP3-GSDMD signaling; AMPK signaling; Caspase-3 signaling; NF-κB signaling | [1-4] |
| Endocrine system | Thyroid dysfunction; Growth inhibition | DNA damage; GH/IGF-1 axis disruption; HPT axis disruption; Adipogenic disruption | IRS1-PI3K-Akt signaling | [5, 6] |
| Circulatory system | Atherosclerosis; Myocardial dysplasia | ER stress; Cardiac fibrosis; Myocardium apoptosis; Cardiac pyroptosis | BMP signaling; NF-κB-NLRP3-GSDMD signaling; AMPK-PGC1α signaling; PERK-eIF2α-ATF4 signaling; LC3-p62 signaling; Wnt/β-catenin signaling | [7-11] |
| Respiratory system | Pulmonary fibrosis; Asthma; COPD | Inflammation; Oxidative stress; Mitochondrial dysfunction; Extracellular matrix overproduction | IL-8 signaling; p38 signaling; NF-κB signaling; Wnt/β-catenin signaling | [12-14] |
| Locomotor system | Rheumatoid arthritis; Osteoporosis | Inflammation; Cartilage erosion; Macrophage infiltration; Osteoclast differentiation; RANKL/OPG axis disruption | p38 signaling; NF-κB signaling; RANK-NFATc1 signaling | [15, 16] |
| Immune system | Autoimmune diseases; Tumors | T-cell exhaustion; Oxidative stress; DNA damage | JAK-STAT signaling; NF-κB signaling; NLRP3-IL-1β signaling | [17, 18] |
| Digestive system | Colitis | Inflammation; Gut microbiota dysbiosis; Increased gut permeability | NLRP3/Caspase-1 signaling; Wnt/β-catenin signaling | [19, 20] |
| Urinary system | Chronic kidney disease | Oxidative stress; Necroptosis; ER stress; Autophagy | Nrf2 signaling; NF-κB signaling; RIP1-RIP3-MLKL signaling; IRE1α-XBP1 signaling; AMPK-ULK1 signaling | [21-23] |
| Reproductive system | Female infertility; Male infertility | Oxidative stress; Apoptosis; DNA damage; Autophagy | Hippo signaling; PI3K-Akt signaling; FOXA1/MAP3K1/p38 signaling | [24-26] |

**References**

1. Jung, Byung-Kwon, Seung-Woo Han, So-Hyun Park, Jin-Sil Bae, Jinhee Choi, Kwon-Yul Ryu. 2020. “Neurotoxic potential of polystyrene nanoplastics in primary cells originating from mouse brain.” *NeuroToxicology* 81: 189–196. https://doi.org/10.1016/j.neuro.2020.10.008
2. Schirinzi, Gabriella F., Ignacio Pérez-Pomeda, Josep Sanchís, Cesare Rossini, Marinella Farré, Damià Barceló. 2017. “Cytotoxic effects of commonly used nanomaterials and microplastics on cerebral and epithelial human cells.” *Environmental Research* 159: 579–587. https://doi.org/10.1016/j.envres.2017.08.043
3. Sun, Meng, Min Zhang, Fanglin Di, Weijie Bai, Jikui Sun, Mingkun Zhang, Jinlong Sun, et al. 2024. “Polystyrene nanoplastics induced learning and memory impairments in mice by damaging the glymphatic system.” *Ecotoxicology and Environmental Safety* 284: 116874. https://doi.org/10.1016/j.ecoenv.2024.116874
4. Yin, Kai, Hongmin Lu, Yue Zhang, Lulu Hou, Xin Meng, Junbo Li, Hongjing Zhao, et al. 2022. “Secondary brain injury after polystyrene microplastic-induced intracerebral hemorrhage is associated with inflammation and pyroptosis.” *Chemico-Biological Interactions* 367: 110180. https://doi.org/10.1016/j.cbi.2022.110180
5. Ge, Qing, Tong Zheng, Ping Ding, Zhenyuan Li, Xihua Lin, Xintong Li, Miao He, et al. 2025. “Aged microplastics-induced growth inhibition via DNA damage, GH/IGF-1 and HPT axes disruption in zebrafish larvae.” *Science of The Total Environment* 975: 179215. https://doi.org/10.1016/j.scitotenv.2025.179215
6. Moon, Hanbyeol, Damin Jeong, Jung-Won Choi, Seongtae Jeong, Hojin Kim, Byeong-Wook Song, Soyeon Lim, et al. 2024. “Microplastic exposure linked to accelerated aging and impaired adipogenesis in fat cells.” *Scientific Reports* 14: 23920. https://doi.org/10.1038/s41598-024-74892-6
7. Li, Zekang, Shuxiang Zhu, Qian Liu, Jialiu Wei, Yinchuan Jin, Xifeng Wang, Lianshuang Zhang. 2020. “Polystyrene microplastics cause cardiac fibrosis by activating Wnt/β-catenin signaling pathway and promoting cardiomyocyte apoptosis in rats.” *Environmental Pollution* 265: 115025. https://doi.org/10.1016/j.envpol.2020.115025
8. Wei, Jialiu, Xifeng Wang, Qian Liu, Na Zhou, Shuxiang Zhu, Zekang Li, Xiaoli Li, et al. 2021. “The impact of polystyrene microplastics on cardiomyocytes pyroptosis through NLRP3/Caspase-1 signaling pathway and oxidative stress in Wistar rats.” *Environmental Toxicology* 36: 935–944. https://doi.org/10.1002/tox.23095
9. Yang, Na, Bo Wu, Xiaoxue He, Junhu Ma, Longhao Dai, RuiTing Ma, Tingting Yang, et al. 2025. “Polystyrene bead ingestion promotes atherosclerosis plaque progression via BMP signaling in mice.” *Food and Chemical Toxicology* 202: 115455. https://doi.org/10.1016/j.fct.2025.115455
10. Zhang, Yue, Dongxu Wang, Kai Yin, Hongjing Zhao, Hongmin Lu, Xin Meng, Lulu Hou, et al. 2022. “Endoplasmic reticulum stress-controlled autophagic pathway promotes polystyrene microplastics-induced myocardial dysplasia in birds.” *Environmental Pollution* 311: 119963. https://doi.org/10.1016/j.envpol.2022.119963
11. Zhang, Yue, Kai Yin, Dongxu Wang, Yu Wang, Hongmin Lu, Hongjing Zhao, Mingwei Xing. 2022. “Polystyrene microplastics-induced cardiotoxicity in chickens via the ROS-driven NF-κB-NLRP3-GSDMD and AMPK-PGC-1α axes.” *Science of The Total Environment* 840: 156727. https://doi.org/10.1016/j.scitotenv.2022.156727
12. Li, Xuran, Tongtong Zhang, Wenting Lv, Hui Wang, Haoran Chen, Qinghua Xu, Hourong Cai, et al. 2022. “Intratracheal administration of polystyrene microplastics induces pulmonary fibrosis by activating oxidative stress and Wnt/β-catenin signaling pathway in mice.” *Ecotoxicology and Environmental Safety* 232: 113238. https://doi.org/10.1016/j.ecoenv.2022.113238
13. Paplińska-Goryca, Magdalena, Paulina Misiukiewicz-Stępień, Monika Wróbel, Katarzyna Mycroft-Rzeszotarska, Dorota Adamska, Julia Rachowka, Milena Królikowska, et al. 2025. “The impaired response of nasal epithelial cells to microplastic stimulation in asthma and COPD.” *Scientific Reports* 15: 4242. https://doi.org/10.1038/s41598-025-87242-x
14. Woo, Jong-Hwan, Hyeon Jin Seo, Jun-Young Lee, Iljung Lee, Kisoo Jeon, Bumseok Kim, Kyuhong Lee. 2023. “Polypropylene nanoplastic exposure leads to lung inflammation through p38-mediated NF-κB pathway due to mitochondrial damage.” *Particle and Fibre Toxicology* 20: 2. https://doi.org/10.1186/s12989-022-00512-8
15. Lee, Su-Hyun, Gi Heon Jeong, Min-Kyung Nam, Moon Hwa Kwak, Chaerin Kim, Se-Hyeon Park, Jiyoung Yeo, et al. 2025. “Polystyrene microplastics activate NF-κB/MAPK signaling in synovial fibroblasts, promoting inflammation and joint destruction in rheumatoid arthritis.” *Journal of Hazardous Materials* 499: 140194. https://doi.org/10.1016/j.jhazmat.2025.140194
16. Zhang, Weilin, Kuize Liu, Boyuan Zhou, Dao Feng, Zhencong Li, Zhiwen Dai, Shengbang Huang, et al. 2026. “RANKL/OPG axis as a therapeutic target for microplastic-induced bone loss: Mechanistic insights from transcriptomic and functional validation.” *Toxicology Letters* 415: 111789. https://doi.org/10.1016/j.toxlet.2025.111789
17. Chen, Jin-Can, Chao Fang, Rong-Hui Zheng, Ming-Liang Chen, Duck-Hyun Kim, Young-Hwan Lee, Christyn Bailey, et al. 2022. “Environmentally relevant concentrations of microplastics modulated the immune response and swimming activity, and impaired the development of marine medaka *Oryzias melastigma* larvae.” *Ecotoxicology and Environmental Safety* 241: 113843. https://doi.org/10.1016/j.ecoenv.2022.113843
18. Zhao, Jun, Haibo Zhang, Lei Shi, Yongshi Jia, Hailong Sheng. 2024. “Detection and quantification of microplastics in various types of human tumor tissues.” *Ecotoxicology and Environmental Safety* 283:116818. https://doi.org/10.1016/j.ecoenv.2024.116818
19. Deng, Yongfeng, Hexia Chen, Yichao Huang, Yan Zhang, Hongqiang Ren, Mingliang Fang, Qing Wang, et al. 2022. “Long-term exposure to environmentally relevant doses of large polystyrene microplastics disturbs lipid homeostasis via bowel function interference.” *Environmental Science & Technology* 56: 15805–15817. https://doi.org/10.1021/acs.est.1c07933
20. Li, Qi, Xiaofeng Wang, Yaqing Xu, Wuqiong Zhang, Yingnan Liu, Su Li, Xiaoman Suo, et al. 2026. “Trojan horse effect of biologically aged microplastics-An intracellular carrier of LPS for licensing noncanonical inflammasome activation.” *Environmental Science & Technology* 60: 281–298. https://doi.org/10.1021/acs.est.5c12882
21. Huang, Hang, Pengyu Lei, Haiyang Yu, Jiao Du, Baihui Wu, Hanbing Wang, Qinsi Yang, et al. 2024. “Micro/nano plastics in the urinary system: Pathways, mechanisms, and health risks.” *Environment International* 193: 109109. https://doi.org/10.1016/j.envint.2024.109109
22. Meng, Xin, Kai Yin, Yue Zhang, Dongxu Wang, Hongmin Lu, Lulu Hou, Hongjing Zhao, et al. 2022. “Polystyrene microplastics induced oxidative stress, inflammation and necroptosis via NF-κB and RIP1/RIP3/MLKL pathway in chicken kidney.” *Toxicology* 478: 153296. https://doi.org/10.1016/j.tox.2022.153296
23. Sun, Xinyue, Wenyue Zhang, Yuqi Wang, Yilei Zhang, Xiaojing Liu, Xu Shi, Shiwen Xu. 2023. “Combined exposure to di(2-ethylhexyl) phthalate and polystyrene microplastics induced renal autophagy through the ROS/AMPK/ULK1 pathway.” *Food and Chemical Toxicology* 171: 113521. https://doi.org/10.1016/j.fct.2022.113521
24. Qu, Jiayuan, Jiayue Zeng, Li Mou, Xiaobin Wu, Mei Ha, Changjiang Liu. 2025. “Plastic tableware use, microplastic accumulation, and sperm quality: From epidemiological evidence to FOXA1/p38 mechanistic insights.” *Journal of Nanobiotechnology* 23: 634. https://doi.org/10.1186/s12951-025-03747-7
25. Zeng, Lianjie, Chong Zhou, Wenqing Xu, Yupei Huang, Wencan Wang, Zhangqiang Ma, Jian Huang, et al. 2023. “The ovarian-related effects of polystyrene nanoplastics on human ovarian granulosa cells and female mice.” *Ecotoxicology and Environmental Safety* 257: 114941. https://doi.org/10.1016/j.ecoenv.2023.114941
26. Zhang, Chenming, Xiaofei Han, Yifei Wang, Ruimin Ma, Sicheng Ma, Wenbang Liu, Zhe Chang, et al. 2026. “The mouse model of induced sperm DNA damage caused by polystyrene microplastics exhibited distinct transcriptomic and proteomic features.” *Reproductive Biology* 26: 101096. https://doi.org/10.1016/j.repbio.2025.101096
